# Standard Reference Materials for Cement Paste: Part III—Analysis of the Flow Characteristics for the Developed Standard Reference Material According to Temperature Change

**DOI:** 10.3390/ma11102001

**Published:** 2018-10-16

**Authors:** Dong Kyu Lee, Myoung Sung Choi

**Affiliations:** Department of Safety Engineering, College of Engineering, Dongguk University-Gyeongju, 123 Dongdae-ro, Gyeongju 38066, Korea; dklee@dongguk.ac.kr

**Keywords:** temperature, standard reference materials, rheology, yield stress, plastic viscosity

## Abstract

For the general quantitative evaluation of the flow characteristics of a material, various factors that affect the flow should be examined. Notably, cement paste shows multi-dimensional flow characteristics owing to not only its inherent features such as its various particle sizes and hydration reaction, but also due to environmental factors including temperature, humidity, and pressure. Therefore, an analysis of those environmental factors is important for the quantitative evaluation of the flow characteristics of cement paste. In this study, we analyzed the flow characteristics of cement paste and a newly developed standard reference material (SRM) that has similar flow characteristics to cement paste at different temperatures. For the analysis, the flow characteristics of each cement paste mixture at five different temperatures (5 °C, 10 °C, 20 °C, 30 °C, and 40 °C) were examined, in consideration of variations in construction environments. Then, the flow characteristics of the developed SRM at different temperatures were also analyzed. The result of the analysis demonstrated a decrease in the rheology constant value following a rise in the temperature. Notably, the degree of variation in the flow characteristics was larger at a lower temperature, while flow characteristics remained nearly constant at higher temperatures. The result of the analysis also confirmed that cement paste and the newly developed SRM displayed similar tendencies for the change in flow characteristics following a change in temperature. In conclusion, the newly developed SRM is thought to be useful for consistently representing the flow characteristics of cement paste under various construction environments in consideration of temperature change.

## 1. Introduction

Rheology generally refers to the quantitative analysis of the flow condition of a material, and is utilized in diverse fields including polymers [1], food [2], biology [3], chemistry [4], and lubricants [5]. Recently, more high-rise buildings have been built based on advancements in the construction industry and owing to a lack of available land. In addition, the demand for buildings with complex structures has increased in line with the development of the architectural design industry. Accordingly, the demand for special concrete structures is increasing, and new, more advanced construction technologies are required [6,7]. Under such circumstances, the concept of rheology has been introduced for the quantitative evaluation of flow characteristics in the field of concrete [8,9,10,11,12,13,14,15,16,17,18,19,20,21,22]. The quality of the concrete structure is, of course, dependent on the quality of each constituent used in the concrete mix. However, this is not the only controlling factor. The quality also depends very much on the rheological properties of the fresh concrete during the placement into the formwork. That is, the concrete must be able to properly flow into all corners of the mold or formwork to fill it completely, with or without external consolidation. Therefore, one of the primary criteria for a good concrete structure is that the fresh concrete has satisfactory rheological properties during casting. Pumping, spreading, molding, and compaction all depend on rheology and thanks to an increasingly scientific approach it is becoming possible to predict fresh properties and design and select materials and model processes to achieve the required performance [9,10]. As such, diverse studies about the quantitative evaluation of construction technology have been conducted recently [11,12,13]. Notably, many studies have been actively conducted for the purpose of developing quantitative evaluation technology by utilizing the concept of rheology, exceeding past methods of comparing the physical characteristics of construction materials relatively [14,15,16,17].

Concrete contains particles of various sizes from cement to coarse aggregate whose particle sizes vary from micron-sized to tens of millimeters [18,19]. In addition, concrete displays multi-dimensional flow characteristics owing to the chemical reaction between cement and moisture (the hydration reaction) [20,21]. As the construction industry expands, more uncertainties arise in quantitatively evaluating the flow characteristic of construction materials, because of the differences in the environments of the construction sites relating to temperature, humidity, and pressure. These factors should be considered when quantitatively evaluating the flow characteristics of concrete. However, it is difficult to evaluate the flow characteristics due to the variation of material properties according to the manufacturing process and its multi-dimensional properties. For this reason, some studies have been conducted on the development of standard reference materials (SRM) whose properties are independent of the hydration reaction and factors of the surrounding environment, and that can simulate the flow characteristics of concrete while maintaining consistent quality and performance [16,17,22].

The National Institute of Standards and Technology (NIST) have been actively conducting studies to develop SRM that can be a substitute for concrete. In the first phase of the study, an SRM of cement paste was developed based on various experimental analyses [22]. However, it was uncertain whether its practical use, including a clear definition of its standard form and application, would be possible [22,23,24]. Then, some experimental studies were conducted in Korea by Lee et al. [16,17] to clearly define the SRM, resulting in a more detailed composition of the SRM being drawn, and an SRM production manual for different mixing ratios was developed for the application and commercialization of the SRM. However, the manual was not developed to reflect the diverse environmental conditions of construction sites, meaning it did not consider the differences in environmental factors. Therefore, a further review of the manual in terms of variation in the environment of each construction site is required.

Preceding studies have identified that changes in the temperature of the cement paste (a fundamental component of concrete) determines its overall flow performance and affects the filling decisions, such as the hydration speed and the gap between particles [25,26,27,28,29,30]. Namely, the temperature has multi-dimensional influences on the evaluation of the flow characteristics of concrete. Therefore, in this study, the flow characteristics of a developed SRM based on the existing rheology characteristics at different temperatures was analyzed to evaluate the application performance as a means to represent the flow characteristics of cement paste.

## 2. Experimental Design and Method

### 2.1. Experimental Design

To develop an SRM, it is necessary to first analyze its conformance with the requirements for SRMs. There are five requirements for SRMs containing particles, which are currently suggested based on preceding international studies: particle separation should not take place while the experiment is ongoing; the linear Bingham reaction should be shown at a large-scope shear rate; no rheological or chemical change should occur between the fluid and particles over a long period of time; the yield stress should be sufficient to prevent a separation between the materials of the aggregate; and double-sided linear response behavior (hysteresis) should not occur [16,17,22]. The components of SRM for cement paste—limestone, glycerol, and distilled water—were used in this study [16]. This composition fulfilled all the requirements for an SRM. Limestone showed little reaction to the moist conditions and since its mean grain diameter was similar to that of cement, it was selected as a substitute for cement powder. In addition, glycerol and distilled water which have properties similar to the flow performance properties of cement paste matrix, as well as chemical stability showing a consistent viscosity with time, were selected as substitute for the matrix fluid.

**Figure 1:**
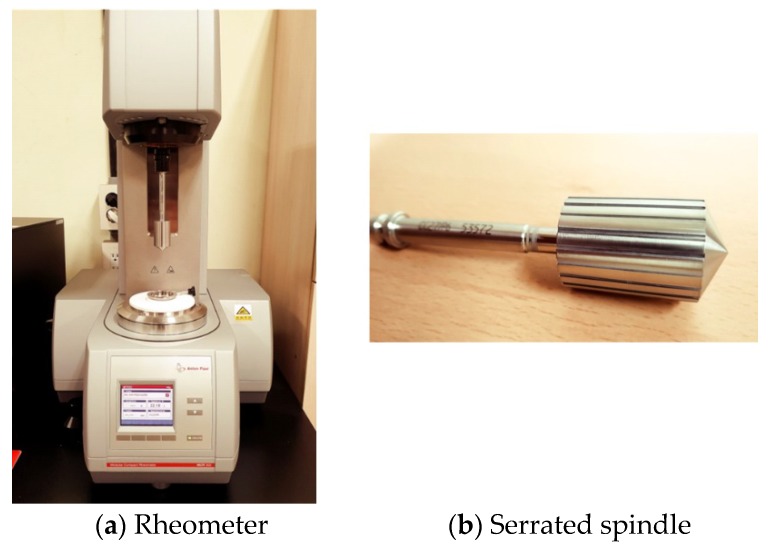
The rheology measurement system.

A parallel plate measurement system with a serrated spindle with a 50-mm diameter was used as the rheometer measurement system in preceding studies [16,17]. As a small amount of material (3 g approximately) was put into the measurement system, the rheology measurement value was not rendered consistently owing to factors such as evaporation of moisture from the surface of the material following a rise in the temperature. Therefore, this study used a concentric cylinder measurement system that is not accompanied by a change in the physical properties of the material caused by the evaporation of moisture following a change in temperature, due to a sufficient quantity of material being used (Figure 1a). In addition, a serrated spindle was used to prevent the slip phenomenon (Figure 1b). The concentric cylinder measurement system was used based on the results of previous studies that showed that the value is affected by the amount of limestone; plastic viscosity (by the ratio of glycerol and distilled water); and hysteresis (by the amount of glycerol). First, the mixture ratio of an SRM according to the mixture ratio of cement paste at room temperature (20 °C) was determined using Equations (1) and (2), as follows [17]:

(1) W/L=0.8×(W/C)+0.0175

(2) G/L=0.09

The experiments in this study were conducted under five temperature conditions (5 °C, 10 °C, 20 °C, 30 °C, and 40 °C), in consideration of international construction environments. The flow characteristics for each temperature according to W/C were first analyzed. Based on these results, the flow characteristics of different mixing ratios of the standard reference material drawn from Equations (1) and (2) for each temperature were analyzed.

### 2.2. Experiment Method

The rheometer (Anton-Paar) used in this study can measure the flow characteristics of materials quantitatively, and the temperature for measurement can be set (−35 °C to +200 °C) directly by users. Flow characteristics are generally determined from the relationship between the shear stress and shear rate applied to the material. The Bingham model Equation (Equation (3)) was used to determine the plastic viscosity and yield stress [31,32,33,34,35,36]. The plastic viscosity is defined as the shear stress–shear rate inclination, and the yield stress is the *y*-intercept drawn through regression analysis [37,38].

(3) $$\tau =\eta \dot{\gamma }+\tau_{0}$$

where *τ* = shear stress, *η* = plastic viscosity, $\dot{\gamma }$ = shear rate, *τ*_0_ = yield stress.

The material for the experiment was prepared to reach the set temperature by using a thermos-hygrostat (−20 °C to +100 °C) for approximately 30 min to minimize the time consumed by the material to reach the set temperature. The rheometer was set to reach the targeted temperature for the experiment. For the mixture, a high-speed mixer was used to mix the material at the end of each of four stages totaling 120 s (15 s, 15 s, 30 s, 60 s), to obtain consistent properties of the material.

An adequate time was given (50 s^−1^/120 s) before the experiment, for the purpose of homogenizing the material and allowing it to reach the set temperature. Then, a rest time (10 s) was given to suppress the orientation of the material. In addition, the shear resistance applied on the spindle due to the rotating speed was measured. This was set to 10 stages on the upward and downward curve of the shear velocity, increased from 0.1 to 40 s^−1^, and then reduced again to 0.1 s^−1^.

## 3. Results of Rheology for Each Material at Different Temperatures

### 3.1. Results of Rheology for Cement Paste at Different Temperatures

**Figure 2:**
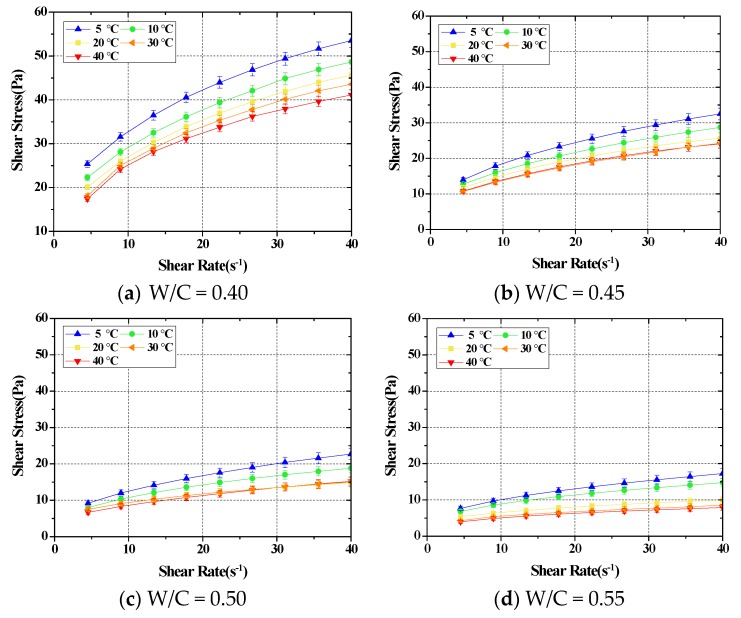
The test results of the rheological properties for different cement paste mixing ratios.

We analyzed the flow characteristics of cement paste mixed in different mixing ratios and the SRM mixed in corresponding ratios, according to changes in the temperature. First, the flow characteristics of cement paste following a change in temperature were analyzed. The cement pastes mixed in four different ratios were tested for analysis of rheology at different temperatures. The values of the flow characteristics of the cement paste showed rather uncertain and multi-dimensional properties owing to various environmental factors and the factors of the material itself, such as the hydration reaction. To solve this issue, the time from the mixing to reaching the set temperature was maintained for 5 min for repeated experiments to obtain an average value [38,39,40,41,42,43,44]. The result is demonstrated in Figure 2.

As shown in the results of the experiment, the plastic viscosity and yield values of cement pastes in all mixing ratios displayed tendencies to decrease following a rise in temperature. The change rate of the entire plastic viscosity was approximately 33%–53% at low temperatures of 5 °C and 10 °C, and approximately 0%–12% at higher temperatures of 30 °C and 40 °C. The entire change rate of the yield stress was approximately 14%–65% at lower temperatures and 7%–13% at higher temperatures. In other words, a change in flow characteristics following temperature change takes place mostly at lower temperatures, while flow characteristics are maintained at a similar level at higher temperatures.

Generally, the decrease of yield stress at high temperatures (30 °C and 40 °C) can be attributed to an increase in the Brownian motion of the particles, which partially weakens the interactions between agglomerates. In addition, this thermal agitation is also favored by the decrease in the viscosity of the continuous phase. Moreover, it is known that as the temperature increases, the interactions and bonding force between the particles weaken, the volume increases as the intergranular distance increases, and thus the viscosity decreases. The shear thinning phenomenon is known to be influenced by the size and shear rate of the particles. Especially, as the shear rate increases, it is known that the number of collisions between particles is gradually reduced in the direction of shearing, gradually decreasing from the equilibrium state, thereby keeping the viscosity constantly decreased. Similarly, at low temperatures, the bonding force between the particles is high so that the number of collisions between the particles is high, which results in a high viscosity value.

**Figure 3:**
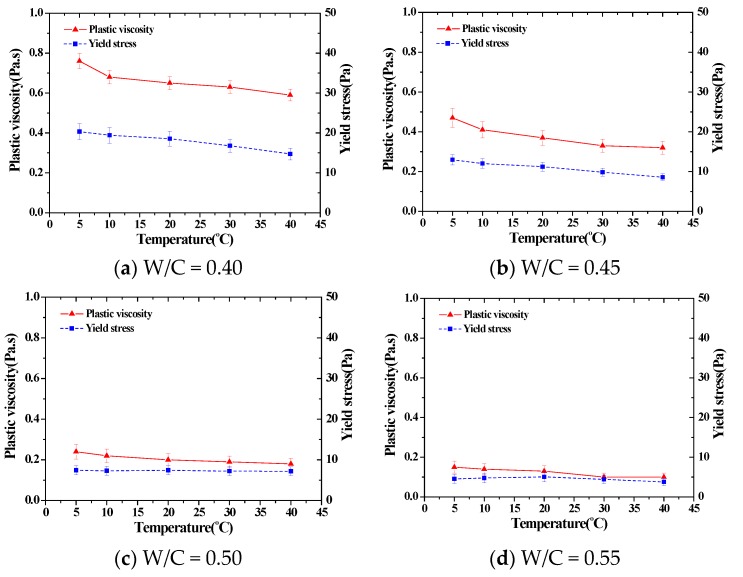
The rheological parameters of different cement pastes by mixing ratios.

Figure 3 is a graph that shows the tendency of the change in flow characteristics following a temperature change in each mixture of cement paste. Table 1 is a summary of Figure 3, as mentioned above, which shows that changes in flow characteristics occur at lower temperatures, but a constant rheology value is maintained at higher temperatures in the range of mixtures used.

### 3.2. Results of Rheology for Standard Reference Materials at Different Temperatures

In the next stage of the analysis, flow characteristics according to temperature change for the developed SRM that can simulate the flow characteristics of the cement pastes of different mixing ratios drawn from Equations (1) and (2) were analyzed.

As mentioned above, the derived Equations (1) and (2) are different from the previous research due to different measurement systems. However, from the previous research, we have known that the water-limestone ratio affects the yield value and plastic viscosity, glycerol-limestone ratio affects hysteresis using Equation (4) below [17]. More detailed information could be found in Reference [17].

(4) $$\frac{W+G}{L}=\frac{W}{L}+\frac{G}{L}$$

**Figure 4:**
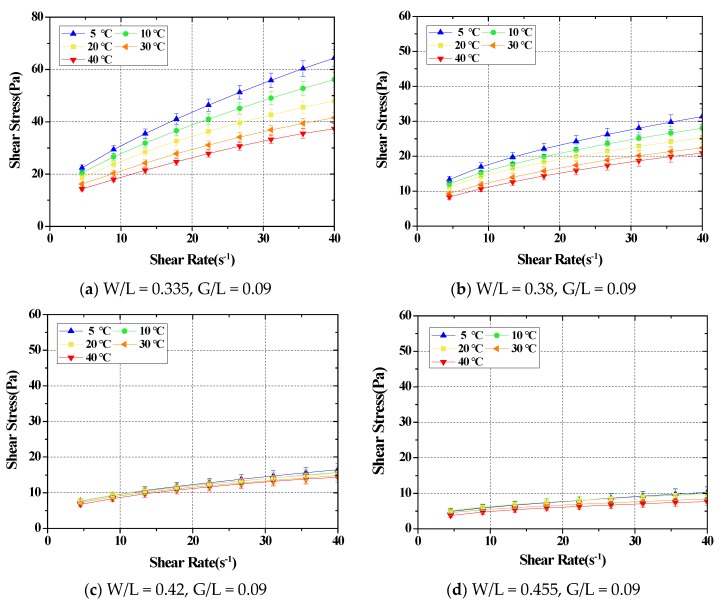
The test results of the rheological properties for standard reference materials by mixing ratios.

The 5 min from mixing to reaching the setting temperature was applied to conduct the experiment on SRM under the same conditions as the experiment for cement paste. Figure 4 shows the results of a rheology analysis for SRMs mixed in different ratios following a temperature change.

**Figure 5:**
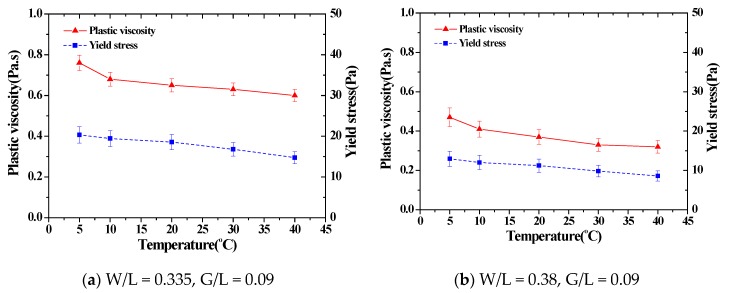

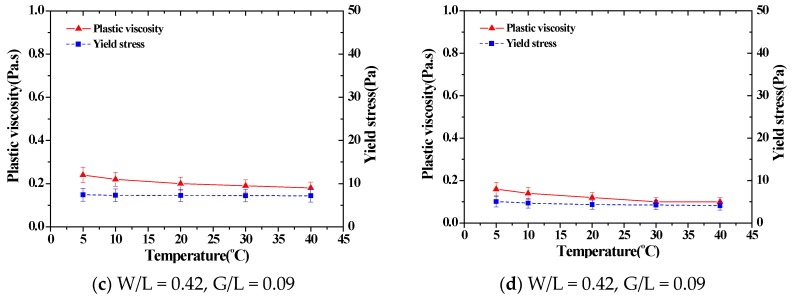
The rheological parameters for standard reference materials by mixing ratios.

Changes in the flow characteristics of SRM following a temperature change were analyzed. In the results, the rheology constant value showed a tendency to decrease following an increase in temperature for all mixtures, similar to the cement paste. In addition, the recorded change rate of the plastic viscosity in each mixing ratio was approximately 20%–35% at lower temperatures of 5 °C and 10 °C, and 0%–19% at higher temperatures of 30 °C and 40 °C. The recorded change rates of all yield values were 16%–52% at lower temperatures, and 9%–36% at higher temperatures. In short, changes in the flow characteristics of the SRM following a temperature change mostly occurred at lower temperatures, similar to cement paste. The change in the flow characteristics maintained a similar level at higher temperatures. The graph in Figure 5 displays the tendency of the change in plastic viscosity and yield value following a temperature change. As mentioned previously, changes in flow characteristics took place at lower temperatures for all mixtures, but plastic viscosity and yield stress maintained a constant level at higher temperatures (Table 2).

### 3.3. Comparison of Flow Characteristics of Cement Paste and SRM at Different Temperatures

**Figure 6:**
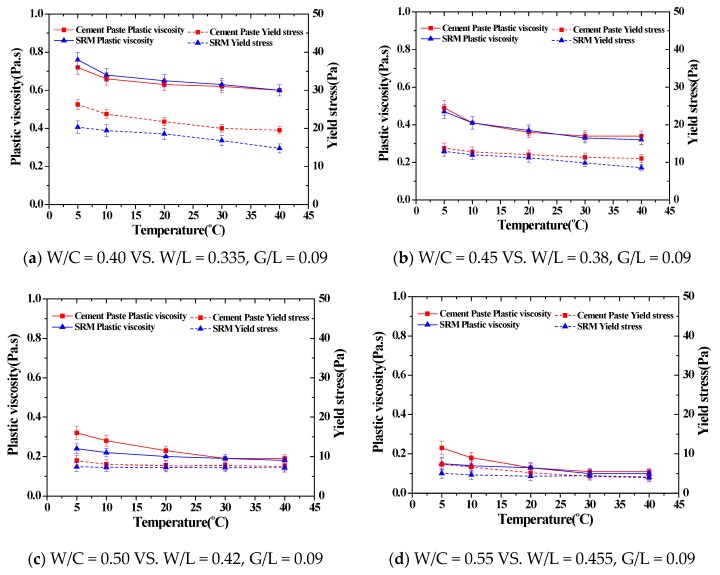
The comparison of the rheological properties of cement paste and standard reference materials.

The result of the rheology analysis on each cement paste mixture at different temperatures was compared with the result of rheology analysis on each mixture of SRM. As shown in Figure 6, all mixtures of cement paste and newly developed SRM displayed similar changes in flow characteristics at each temperature applied to the experiment. These results indicate that the newly developed SRM sufficiently simulates the flow characteristics of cement paste at different temperatures. Therefore, the newly developed SRM is thought to be useful as an SRM that can represent certain flow characteristics in diverse construction conditions, as the level of its sensitivity to temperature is low.

## 4. Discussion

As shown in Figure 6a, there is a slight difference in the yield stress values because the behavior of the particles varies with the changes in measurement systems which could cause some errors. So, the mixing ratio of the standard reference material corresponding to each combination of cement paste was newly derived based on a new measurement system. Here, the minimum amount of glycerol that does not cause hysteresis in the modified measurement system was analyzed to be 9% of the amount of limestone, and the standard reference material combination that can best simulate the W/C = 0.40 combination was W/L = 0.335, G/L = 0.09. In the case of the cement paste, when the flow rate of the cement paste is measured, normally there is an error of ±3 Pa in the yield value. However, in order to reduce the difference in the yield values shown in Figure 6a, the addition of the limestone amount can cause a slight difference in plastic viscosity. In other words, it is considered that the research on the direction of increasing the yield value and decreasing the plastic viscosity should be necessary while controlling the amount of glycerol to prevent the occurrence of hysteresis. As an alternative to this, the relationship between the yield values measured in the rheological analysis for each measurement system can be established as a formula, and a study can be conducted to derive the difference of the yield value without changing the compounding ratio of the standard reference materials for each measurement system. It is expected that it will be necessary to carry out additional research.

## 5. Conclusions

In this study, based on the previous research which determined the composition of an SRM that can simulate the flow characteristics of cement paste, the factors related with temperature that operate as the largest variables in the applicability and commercialization of an SRM were studied. The results of this study are as follows:

1. Based on the previous study, it was known that the ratio of W/L affects the yield and plastic viscosity and the G/L affects the hysteresis, so by using these relations, we have proposed new formula because measurement systems are changed.
2. In consideration of construction environment, changes in the flow characteristics of each mixture of cement paste at five different temperatures (5 °C, 10 °C, 20 °C, 30 °C, and 40 °C) were analyzed. For all the mixtures, the flow characteristics of cement paste tended to decrease following a rise in temperature. Slight changes occurred in the flow characteristics at lower temperatures, but the degree of change was reduced following a rise in temperature, and a nearly constant rheology value was maintained.
3. The decrease of the yield stress at high temperatures (30 °C and 40 °C) can be attributed to an increase in the Brownian motion of the particles, which partially weakens the interactions between agglomerates. In addition, this thermal agitation is also favored by the decrease in the viscosity of the continuous phase.
4. Moreover, these results indicate that as the temperature increases, the interactions between the particles and the bonding force weaken and the volume increases as the inter-particle distance becomes distant, which leads to a decrease in viscosity. In addition, at low temperatures, the bonding force between the particles is high, and the number of collisions between the particles is high, so they have a high viscosity value.
5. Changes in the flow characteristics in SRM mixtures (corresponding to cement paste mixtures) at five temperatures were analyzed. For all mixtures of the SRM, the degree of change in the flow characteristics demonstrated a tendency to decrease following a rise in temperature, similar to cement paste. The degree of change in flow characteristics was reduced following a rise in temperature, and a consistent rheology value was maintained at higher temperatures.
6. The tendency of changes in the flow characteristics of each mixture of cement paste and SRM following a temperature change was compared. For all mixtures, a similar tendency of change was displayed. At lower temperatures, a high rheology constant value was obtained, but at other temperature conditions, a consistent rheology constant value was obtained.
7. Succinctly, both the cement paste and newly developed SRM demonstrated a tendency of low sensitivity to temperature change in flow characteristics. Such a result indicates the usefulness of the newly developed SRM as a material that can represent a certain flow performance under a construction environment, especially in various temperature conditions.

## Figures and Tables

**Table 1:** The variation of the flow characteristics of cement pastes according to various temperatures.

Temperature (°C)	Cement Paste							
	W/C = 0.40		W/C = 0.45		W/C = 0.50		W/C = 0.55	
	Plastic Viscosity (Pa·s)	Yield Stress (Pa)	Plastic Viscosity (Pa·s)	Yield Stress (Pa)	Plastic Viscosity (Pa·s)	Yield Stress (Pa)	Plastic Viscosity (Pa·s)	Yield Stress (Pa)
5	0.72 ± 0.1	26.23 ± 3	0.49 ± 0.1	13.77 ± 3	0.32 ± 0.1	8.96 ± 3	0.26 ± 0.1	7.35 ± 3
10	0.66 ± 0.1	23.77 ± 3	0.41 ± 0.1	12.81 ± 3	0.28 ± 0.1	8.03 ± 3	0.21 ± 0.1	6.67 ± 3
20	0.63 ± 0.1	21.78 ± 3	0.36 ± 0.1	12.04 ± 3	0.23 ± 0.1	7.68 ± 3	0.13 ± 0.1	5.25 ± 3
30	0.62 ± 0.1	20.02 ± 3	0.34 ± 0.1	11.35 ± 3	0.19 ± 0.1	7.72 ± 3	0.11 ± 0.1	4.29 ± 3
40	0.60 ± 0.1	19.52 ± 3	0.34 ± 0.1	11.02 ± 3	0.19 ± 0.1	7.54 ± 3	0.11 ± 0.1	3.95 ± 3

**Table 2:** The variation of the flow characteristics of SRM according to various temperatures.

Temperature (°C)	SRM							
	W/L = 0.335, G/L = 0.09		W/L = 0.38, G/L = 0.09		W/L = 0.42, G/L = 0.09		W/L = 0.455, G/L = 0.09	
	Plastic Viscosity (Pa·s)	Yield Stress (Pa)	Plastic Viscosity (Pa·s)	Yield Stress (Pa)	Plastic Viscosity (Pa·s)	Yield Stress (Pa)	Plastic Viscosity (Pa·s)	Yield Stress (Pa)
5	0.76 ± 0.1	20.33 ± 3	0.47 ± 0.1	12.96 ± 3	0.24 ± 0.1	7.44 ± 3	0.15 ± 0.1	5.06 ± 3
10	0.68 ± 0.1	19.42 ± 3	0.41 ± 0.1	12.03 ± 3	0.22 ± 0.1	7.31 ± 3	0.14 ± 0.1	4.67 ± 3
20	0.65 ± 0.1	18.56 ± 3	0.37 ± 0.1	11.23 ± 3	0.20 ± 0.1	7.26 ± 3	0.13 ± 0.1	4.32 ± 3
30	0.63 ± 0.1	16.78 ± 3	0.33 ± 0.1	9.83 ± 3	0.19 ± 0.1	7.23 ± 3	0.10 ± 0.1	4.18 ± 3
40	0.60 ± 0.1	14.74 ± 3	0.32 ± 0.1	8.61 ± 3	0.18 ± 0.1	7.19 ± 3	0.10 ± 0.1	4.09 ± 3
